# Bidirectional acoustic negative refraction based on a pair of metasurfaces with both local and global *PT*-symmetries

**DOI:** 10.1038/s41598-020-67793-x

**Published:** 2020-07-01

**Authors:** Jun Lan, Xiaowei Zhang, Liwei Wang, Yun Lai, Xiaozhou Liu

**Affiliations:** 1 Key Laboratory of Modern Acoustics, Institute of Acoustics and School of Physics, Collaborative Innovation Center of Advanced Microstructures, Nanjing University, Nanjing, 210093 People’s Republic of China; 2 Key Laboratory of Modern Acoustics, National Laboratory of Solid State Microstructures, School of Physics, and Collaborative Innovation Center of Advanced Microstructures, Nanjing University, Nanjing, 210093 People’s Republic of China

**Keywords:** Acoustics, Composites

## Abstract

Negative refraction plays an important role in acoustic wave manipulation and imaging. However, conventional systems based on acoustic metamaterials suffer from the limits induced by loss-related and resolution issues. In this work, a parity-time (*PT*)-symmetric system is introduced to realize loss-free bidirectional acoustic negative refraction. The system is composed of a pair of locally *PT*-symmetric multi-layer metasurfaces sandwiching a region of free space, which also forms a global *PT* symmetry. The property of bidirectional negative refraction, which is rare for general *PT*-symmetric structures, is related to the coexistence of amplification and absorption in the locally *PT*-symmetric metasurfaces at their *PT*-broken phases. Such metasurfaces can freely switch their states between coherent perfect absorber (CPA) and amplifier depending on the direction of incidence. Our results provide a physical mechanism for realizing bidirectional functions in acoustic *PT*-symmetric systems.

## Introduction

Veselago used the constitutive parameters of dielectric permeability $\mu$ and magnetic permittivity $\varepsilon$ to define the concepts of single negative and double negative in electromagnetic metamaterial^1^. The unusual phenomenon of negative refraction can be induced by the simultaneously negative values of $\mu$ and $\varepsilon$ [i.e., double negativity (DNG)]^2,3^, which promises a wide range of potential applications such as superlens^4,5^ and illusion optics^6^. Since light and sound are both waves with similar characteristics, tremendous interests have been extracted to the acoustics analogue of DNG materials. Constitutive parameters for acoustic media are mass density $\rho$ and bulk modulus $\kappa$, respectively^7,8^. If these two parameters are both negative, the phase velocity of acoustic wave is also negative and negative refraction can be realized. In previous studies, negative refraction has been realized by the artificial structures for classical acoustic waves, such as metamaterials and phononic crystals^9–11^. However, the realization of negative refraction will inevitably leads to significantly increased sensitivity to losses, which imposes inherent challenges in practical applications.

In recent years, the investigation of electromagnetic *PT* symmetry has provided a new approach to realize negative refraction without the need of negative index materials, which may overcome the limitation of the DNG metamaterial designs^12–16^. The original proposal of negative refraction and imaging by *PT*-symmetric systems is composed of a pair of single-layer metasurfaces exhibiting loss and gain^12–14^. The phenomenon of negative refraction is strictly limited to the case of external incidence on the lossy side. The power flow between the *PT*-symmetric metasurfaces always transfers from the gain side to the lossy side. The physical mechanism behind this unidirectional negative refraction is the asymmetric characteristic of the *PT*-symmetric system near the exceptional point (EP). EP is a singular point in the breaking *PT*-symmetric phase, where the eigenvalues and eigenvectors of the non-Hermitian system coalesce simultaneously^17,18^. In a flurry of researches, many extraordinary phenomena associated with the singular EP have been demonstrated, such as light–light switching^19,20^, unidirectional invisibility^21–24^, unidirectional reflectionlessness^25–27^, teleportation^28^, and impurity-immunity^29^. Besides the EP, there exists another type of singular points denoted as CPA-laser point in the electromagnetic *PT* symmetry, which has recently gained attention owing to its singular characteristics for the *PT*-symmetric system^30–34^. At the CPA-laser point, the eigenvalues go to either zero or infinity, corresponding to two mutually exclusive states, i.e., the coherent perfect absorption mode and the lasing mode. While both perfect absorption and lasing have been individually realized in optical thin structure^35–38^, the coexistence of absorption and lasing in a single structure is unconventional, which is the same for acoustic area. The occurrence of the CPA-laser point in the *PT*-broken phase of the *PT*-symmetric structure provides a possibility to realize it^30,33^.

In this work, we show that bidirectional negative refraction can also be obtained independent of the direction of incidence, by using a *PT*-symmetric system involving both local and global *PT*-symmetries. This system is realized by replacing the original gain and lossy metasurfaces in the unidirectional negative refraction system by a pair of locally *PT*-symmetric multi-layer metasurfaces, which still maintain a global *PT* symmetry. Here, each multi-layer metasurface is constructed by both loss and gain media. The balanced loss-gain *PT*-symmetric condition is satisfied to attain local CPA-saser in the *PT*-broken phase of the locally *PT*-symmetric metasurface. CPA-saser point is the acoustic equivalence of optical CPA-laser point in the *PT*-broken phase, where saser corresponds to optical laser^39^. The globally *PT*-symmetric system, which is composed of two identical locally *PT*-symmetric metasurfaces that can simultaneously behave as a perfect absorber and an amplifier, amazingly realizes the exceptional behavior of bidirectional acoustic negative refraction. Compared to the conventional negative refraction associated with the singular EP, the negative refraction in this work is independent of the incident direction of the acoustic wave. Moreover, we find that this *PT*-symmetric system can be designed to achieve negative bending effects by any desired angle, as well as planar focusing effects with good resolution.

## Results

### Design of the bidirectional acoustic negative refraction system

**Figure 1:**
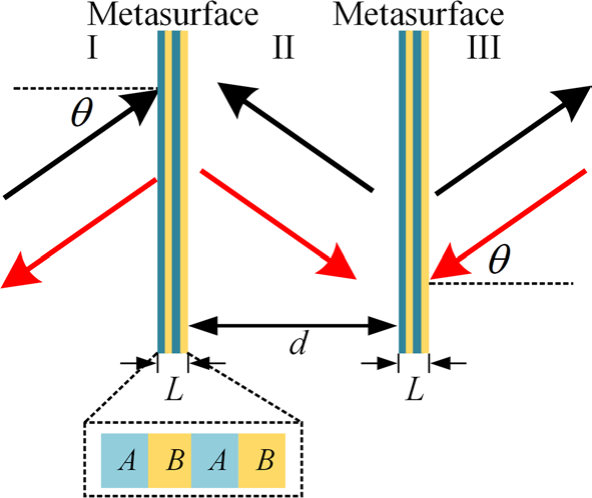
The proposed bidirectional acoustic negative refraction system is composed of a pair of locally *PT*-symmetric multi-layer metasurfaces separated by a free space with distance *d*. The black dotted box marks the arrangement of the loss (*A*) and gain (*B*) layers of the locally *PT*-symmetric metasurface.

The bidirectional acoustic negative refraction system is sketched in Fig. 1, which is a globally *PT*-symmetric system constructed by two identical locally *PT*-symmetric multi-layer metasurfaces, which are separated by a region of free space with distance *d*. Each metasurface is composed of a four-layer structure with loss layers (*A*) and gain layers (*B*) arranged alternatively and periodically. The widths of the loss and gain layers are both one-quarter wavelength *l* = 25 mm and the total width of the metasurface is $L = \lambda_{0}$ ($\lambda_{0}$ is the wavelength in the air). Here, the operating frequency should be equal to the Bragg frequency *f*_*b*_ = 3,430 Hz and the CPA-saser point of the *PT*-symmetric metasurface is approached at this frequency. As shown in Fig. 1, when a plane wave is incident from the left side of *PT*-symmetric system with angle $\theta$ (black arrow in the I region), the multi-reflection between two metasurfaces (II region) forms both forward-traveling and backward-traveling acoustic waves. With appropriate material parameters, the forward-traveling wave can be ignored compared with the backward-traveling wave, and thus the forward-traveling wave is not shown in Fig. 1, as shall be explained later. In this sense, the main energy flux flows from the right side to the left side between two metasurfaces. When the backward-traveling wave transfers to the left metasurface, it will be absorbed by the left metasurface which is under the CPA mode, and there is no reflection in the I region. Meanwhile, when the forward-traveling wave with small value transfers to the right metasurface, it will be transmitted after amplified by the right metasurface. The pressure amplitude of the transmitted wave in the III region is equal to that in the I region, which means the perfect transmission is achieved. Similarly, when a plane wave is incident from the right side of *PT*-symmetric system with angle $\theta$ (red arrow in the III region), the right metasurface absorbs waves from both sides and the left metasurface amplifies the incident wave from II region. Therefore, the bidirectional acoustic negative refraction functionality is obtained as a result of a pair of CPA and amplifier in such a *PT*-symmetric system. At the CPA-saser point, both locally *PT*-symmetric metasurfaces can satisfy functionalities of the amplification and coherent perfect absorption without resorting to altering the frequency or structure.

### Scattering properties

**Figure 2:**
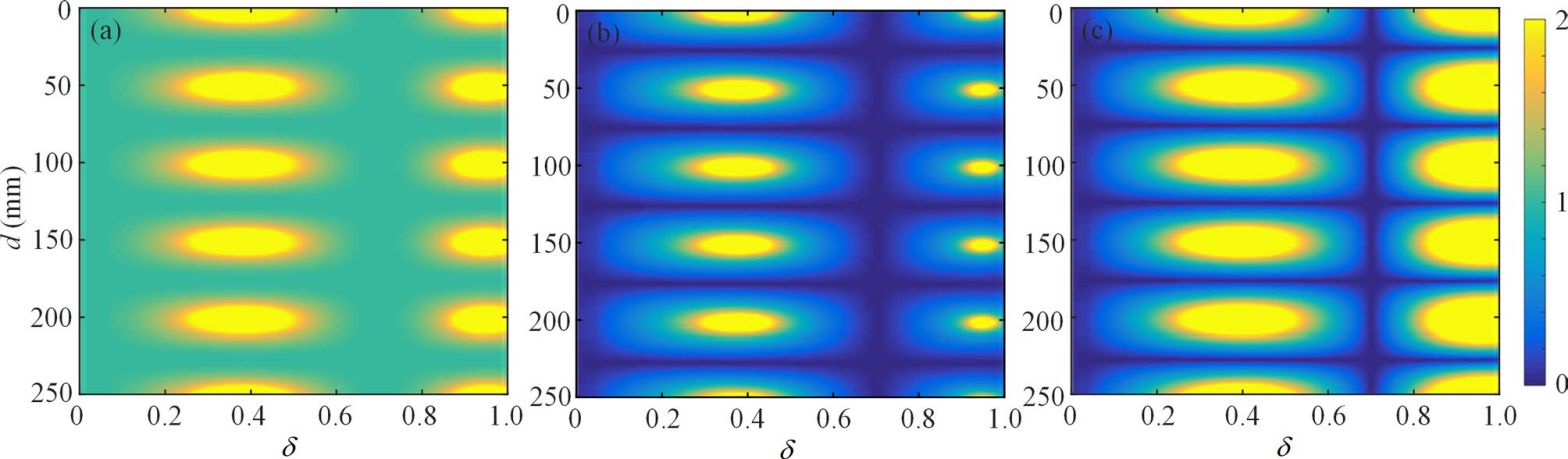
Scattering properties of the globally *PT*-symmetric system. The amplitude of the (**a**) transmission *t*, (**b**) left reflection $r_{L}$, and (**c**) right reflection $r_{R}$ coefficients of the globally *PT*-symmetric system as functions of the values of *d* and $\delta$ for the normally incident wave at the operating frequency of 3,400 Hz.

*PT* symmetry offers an unconventional strategy to utilize loss to control gain, and the intriguing possibility to significantly expand the methods of acoustic wave manipulation^40^. Here, our *PT*-symmetric system is depicted in Fig. 1, which exhibits unique scattering property of bidirectional acoustic negative refraction. To achieve the symmetric responses of amplification and coherent perfect absorption at the same frequency within the locally *PT*-symmetric metasurface, the refractive indices of the loss (*A*) and gain (*B*) layers of the locally *PT*-symmetric metasurface are set as $n_{l} = n_{0} - 0.5\delta i$ and $n_{g} = n_{0} + 0.5\delta i$, respectively, where $n_{0}$ denotes the background refractive index ($n_{0} = 1$) and $\delta$ denotes the loss-gain factor. Both the real and imaginary parts of the refractive index must be in the perfectly balanced condition^30^. The value of $\delta$ will be calculated later. The mass densities of the *A* and* B* layers are set as $\rho_{l} = 1.21\,{\text{kg}}/{\text{m}}^{3}$ and $\rho_{g} = 1.21\,{\text{kg}}/{\text{m}}^{3}$, respectively. For the bidirectional acoustic negative refraction system shown in Fig. 1, the scattering matrix *S* subject to such system with two ports can be expressed as

1 $$\left( {\begin{array}{*{20}c} {p_{bl} } \\ {p_{fr} } \\ \end{array} } \right) = S\left( {\begin{array}{*{20}c} {p_{br} } \\ {p_{fl} } \\ \end{array} } \right) = \left( {\begin{array}{*{20}c} t & {r_{L} } \\ {r_{R} } & t \\ \end{array} } \right)\left( {\begin{array}{*{20}c} {p_{br} } \\ {p_{fl} } \\ \end{array} } \right) = \left( {\begin{array}{*{20}c} {\frac{{t_{1}^{2} E}}{{1 - r_{R1} Er_{L1} E}}} & {\frac{{t_{1}^{2} r_{L1} E^{2} }}{{1 - r_{R1} Er_{L1} E}} + r_{L1} } \\ {\frac{{t_{1}^{2} r_{R1} E^{2} }}{{1 - r_{R1} Er_{L1} E}} + r_{R1} } & {\frac{{t_{1}^{2} E}}{{1 - r_{R1} Er_{L1} E}}} \\ \end{array} } \right)\left( {\begin{array}{*{20}c} {p_{br} } \\ {p_{fl} } \\ \end{array} } \right),$$

where $E = e^{{ - ik_{0} d}}$ and $k_{0}$ is the wave number in free space. $p_{f(b)l}$ and $p_{f(b)r}$ are the components of the forward-(backward-)traveling acoustic wave in the I and III regions, respectively. $r_{L(R)}$ is the left-(right-) reflection coefficient of the globally *PT*-symmetric system. *t* is the transmission coefficient of the globally *PT*-symmetric system, which is identical for both left and right incident waves due to reciprocity. $r_{L1(R1)}$ and $t_{1}$ are the left-(right-) reflection and transmission coefficients of the locally *PT*-symmetric metasurface, respectively. Equation (1) indicates that, for the globally *PT*-symmetric metasurface to be bidirectional and reflectionless, $r_{L1}$, $r_{R1}$ and $t_{1}$ should satisfy $t_{1}^{2} E^{2} /\left( {1 - r_{R1} Er_{L1} E} \right) = - 1$. We start with a design for the case of normal incidence ($\theta = 0$) on the *PT*-symmetric system. The operating frequency is calculated as *f* = 3,400 Hz, which is slightly larger than expected Bragg frequency *f*_*b*_. Based on the transfer matrix method in acoustics (see details in Supplementary Information), the amplitude of the transmission *t*, left reflection $r_{L}$, and right reflection $r_{R}$ coefficients of the globally *PT*-symmetric system as functions of the values of *d* and $\delta$ are calculated, as shown in Fig. 2a–c. It is noted that at the specific value of $\delta = 0.70335$, the perfect transmission (i.e., $\left| t \right| = 1$) and non-reflection (i.e., $\left| {r_{L} } \right| = \left| {r_{R} } \right| = 0$) are obtained for the incidence from the left and right sides, which is independent of the distance *d* between two metasurfaces. In addition, the horizontal blue lines in Fig. 2b,c are induced by the Fabry–Perot resonance of the free space region between two metasurfaces. At the resonant condition for standing-wave excitation (*d* $= (2n - 1)\lambda_{0} /4$, where *n* is an integer), the transmission enhancement and zero reflection can be generated at the two ports of the system^41^. As a result, the proposed *PT*-symmetric system exhibits bidirectional perfect transmission by appropriately selecting the value of $\delta$ or *d*. For the case of $\delta = 0.70335$ and *d* with an arbitrary value, the corresponding scattering matrix of the globally *PT*-symmetric system describing the relationship between the incoming and outgoing waves is given by

2 $$S = \left( {\begin{array}{*{20}c} {e^{{i(k_{0} d\cos \theta + \pi )}} } & 0 \\ 0 & {e^{{i(k_{0} d\cos \theta + \pi )}} } \\ \end{array} } \right),$$

where $\theta$ represents the incident angle. Zero reflection is obtained for the incidence from the left or right side of the system ($r_{L} = r_{R} = 0$), and the transmitted wave undergoes a *phase advance* $(k_{0} d\cos \theta + \pi )$ that is exactly opposite to the one without the pair of *PT*-symmetric metasurfaces. Therefore, when $\delta = 0.70335$, the designed *PT*-symmetric system exhibits loss-free zero reflection, and realizes bidirectional acoustic negative refraction just like a DNG medium of thickness *d* with an additional phase shift $\pi$. The additional phase shift $\pi$ is caused by the *PT*-symmetric metasurfaces. In this case, the scattering matrix describes the fascinating acoustic property of bidirectional acoustic negative refraction, which is rare for general acoustic *PT*-symmetric systems.

The characteristics of the bidirectional non-reflection and phase advance have been guaranteed by Eq. (2), which theoretically confirm that the bidirectional acoustic negative refraction can be successfully achieved by a pair of locally *PT*-symmetric multi-layer metasurfaces. The sound field distributions in the I and III regions shown in Fig. 1 are induced by an acoustic wave propagating from the metasurface far away from the side of incidence, to the metasurface close to the side of incidence, as represented by the black or red arrow (II region). When an acoustic wave is incident from the left side of *PT*-symmetric system, by using a transfer-matrix formalism, the acoustic waves on both I and II regions are connected through a transfer matrix

3 $$\left( {\begin{array}{*{20}c} {p_{fm} } \\ {p_{bm} } \\ \end{array} } \right) = T_{l} \left( {\begin{array}{*{20}c} {p_{fl} } \\ {p_{bl} } \\ \end{array} } \right),$$

where $T_{l}$ and $p_{f(b)m}$ are the transfer matrix and the component of the forward (backward)-traveling acoustic wave in the II region, the details of which are given in Supplementary Eq. (S9). Similarly, when an acoustic wave is incident from the right side of *PT*-symmetric system, the acoustic waves on both II and III regions are connected through a transfer matrix

4 $$\left( {\begin{array}{*{20}c} {p_{fm} } \\ {p_{bm} } \\ \end{array} } \right) = T_{r}^{\prime } \left( {\begin{array}{*{20}c} {p_{fr} } \\ {p_{br} } \\ \end{array} } \right),$$

where $T_{r}^{ \prime }$ is the corresponding transfer matrix, the details of which are given in Supplementary Eq. (S12). For the proposed *PT*-symmetric system, we have obtained that the absolute values of reflection wave $\left| {p_{bl} } \right|$ for left incidence in I region and reflection wave $\left| {p_{fr} } \right|$ for right incidence in III region are almost zero, as shown in Fig. 2b, c. By substituting $\left| {p_{bl} } \right| = 0$ and $\left| {p_{fr} } \right| = 0$ into Eqs. (3) and (4), respectively, the absolute values of the forward-traveling components $\left| {p_{fm} } \right|$ for the acoustic waves incident from the left side and right side cases are $0.0013\left| {p_{fl} } \right|$ and $1.8\left| {p_{br} } \right|$, respectively, and the absolute values of the backward-traveling components $\left| {p_{bm} } \right|$ for the acoustic waves incident from left side and right side cases are $0.556\left| {p_{fl} } \right|$ and $0.0013\left| {p_{br} } \right|$, respectively. Therefore, the propagating direction of the total acoustic wave in the II region is opposite to the direction of the incident acoustic wave in these two cases. Moreover, the relative phase difference between the left incidence in I region (or right incidence in III region) and the total acoustic wave in II region is around $\pi /2$ ($- \pi /2$).

**Figure 3:**
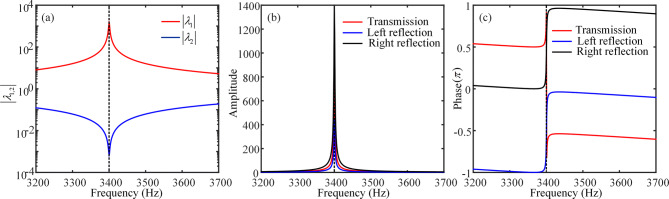
Scattering properties of the locally *PT*-symmetric metasurface. (**a**) The absolute values of two eigenvalues as functions of frequency. (**b**, **c**) The amplitude and phase of the transmission, left-reflection and right-reflection coefficients for the *PT*-symmetric metasurface as functions of frequency, respectively. The vertical dotted lines in (**a**), (**b**) and (**c**) indicate the frequencies of *f* = 3,400 Hz.

This bidirectional characteristic is related to the states of two locally *PT*-symmetric metasurfaces which can uniquely satisfy both the amplification and coherent perfect absorption states at their singular CPA-saser points. In the following, we demonstrate the states of two metasurfaces in the globally *PT*-symmetric system through analyzing the scattering property of the single metasurface. The black dotted box in Fig. 1 has shown the distribution of the loss and gain layers of the *PT*-symmetric metasurface. When $\delta = 0.70335$, the corresponding acoustic velocities in the loss and gain layers are $c_{l} \approx 305.25 + 107.35i$ and $c_{g} \approx 305.25 - 107.35i$, respectively. The transfer matrix method is used to derive the acoustic scattering matrix describing the relationship between the input and output waves, i.e., $$\left( {\begin{array}{*{20}c} {p_{O1} } \\ {p_{O2} } \\ \end{array} } \right) = S_{1} \left( {\begin{array}{*{20}c} {p_{I2} } \\ {p_{I1} } \\ \end{array} } \right) = \left( {\begin{array}{*{20}c} {t_{1} } & {r_{L1} } \\ {r_{R1} } & {t_{1} } \\ \end{array} } \right)\left( {\begin{array}{*{20}c} {p_{I2} } \\ {p_{I1} } \\ \end{array} } \right)$$, where $p_{I(O)1}$ and $p_{I(O)2}$ are the components of the input (output) waves at the left and right ports of the metasurface, respectively. The two eigenvalues of scattering matrix *S*_1_ are expressed as $\lambda_{1,2} = t_{1} \pm \sqrt {r_{L1} r_{R1} }$. Figure 3a presents the absolute values of two eigenvalues $\left| {\lambda_{1,2} } \right|$ as functions of frequency for the locally *PT*-symmetric metasurface. It is seen that, at the operating frequency *f* = 3,400 Hz, the absolute values of two eigenvalues $\left| {\lambda_{1,2} } \right|$ go to either zero or infinity and the local metasurface is in the *PT*-broken phase, which means that the metasurface approaches CPA-saser point. At such a point, the *PT*-symmetric metasurface simultaneously behaves as coherent amplifier and CPA. Moreover, Fig. 3b, c plot the amplitude and phase of the transmission, left reflection and right reflection coefficients of the locally *PT*-symmetric metasurface as functions of frequency, respectively. The results in Fig. 3b show that the amplitude of the transmission, left reflection and right reflection coefficients are all large values ($\left| {t_{1} } \right| \approx 771$, $\left| {r_{L1} } \right| \approx 428.6$ and $\left| {r_{R1} } \right| \approx 1387$), indicating huge scattering is obtained at the CPA-saser point, which means that this metasurface could behave as an amplifier. Figure 3c shows that, at the CPA-saser point, there is a $\pi$ phase difference between two reflections, and the phase differences between the transmission and left- and right-reflections are $\mp \pi /2$. Therefore, in this *PT*-symmetric metasurface, coherent perfect absorption and amplification could be obtained. According to above discussions, the required conditions of the proposed *PT*-symmetric metasurface for coherent perfect absorption and amplification are given by

5 $$\left\{ {\begin{array}{*{20}c} {t_{1} + ar_{L1} e^{i\Delta \phi } = p_{O1} /p_{I2} } \\ {r_{R1} + at_{1} e^{i\Delta \phi } = p_{O2} /p_{I2} } \\ \end{array} } \right.,$$

where $\Delta \phi$ is the relative phase difference between the left and right incident waves, $a = \left| { p_{I1} } \right|/\left| { p_{I2} } \right|$ is the absolute ratio of the left to right incident waves. Equation (5) indicates that, when $a \approx 1.8$ ($\left| {t_{1} } \right|/\left| {r_{L1} } \right| \approx \left| {r_{R1} } \right|/\left| {t_{1} } \right| = 1.8$), the coherent perfect absorption is achieved for $\Delta \phi = \pi /2$. Conversely, the coherent amplification is achieved for $\Delta \phi = - \pi /2$. In addition, we have obtained the pressure field of the forward- and backward-traveling waves in the I, II and III regions of the globally *PT*-symmetric system. When an acoustic wave is incident from the left side of *PT*-symmetric system, for the left metasurface, the ratio of the absolute values of the left to right incident waves (backward-traveling component $p_{bm}$) is $\left| {p_{fl} } \right|/\left| {p_{bm} } \right| \approx 1.8$ (= *a*), and the relative phase difference between the left and right incident waves is $\pi /2$. Thus, the left metasurface acts as a CPA. Meanwhile, the right metasurface behaves as an amplifier. When an incident wave with absolute pressure $\left| {p_{fm} } \right| = 0.0013\left| {p_{fl} } \right|$ is incident on the right metasurface, the absolute pressure of the transmitted wave in the III region is equal to that of the incident wave in the I region, i.e., $\left| {p_{fr} } \right| = \left| {p_{fm} } \right|\left| {t_{1} } \right| = \left| {p_{fl} } \right|$. Besides, when an acoustic wave is incident from the right side of *PT*-symmetric system, the right metasurface acts as a CPA and the left metasurface acts as an amplifier. Therefore, the pair of metasurfaces freely switches the states between CPA-amplifier pair and amplifier-CPA pair dependent on the direction of incidence.

### Bidirectional acoustic negative refraction and planar focusing

**Figure 4:**
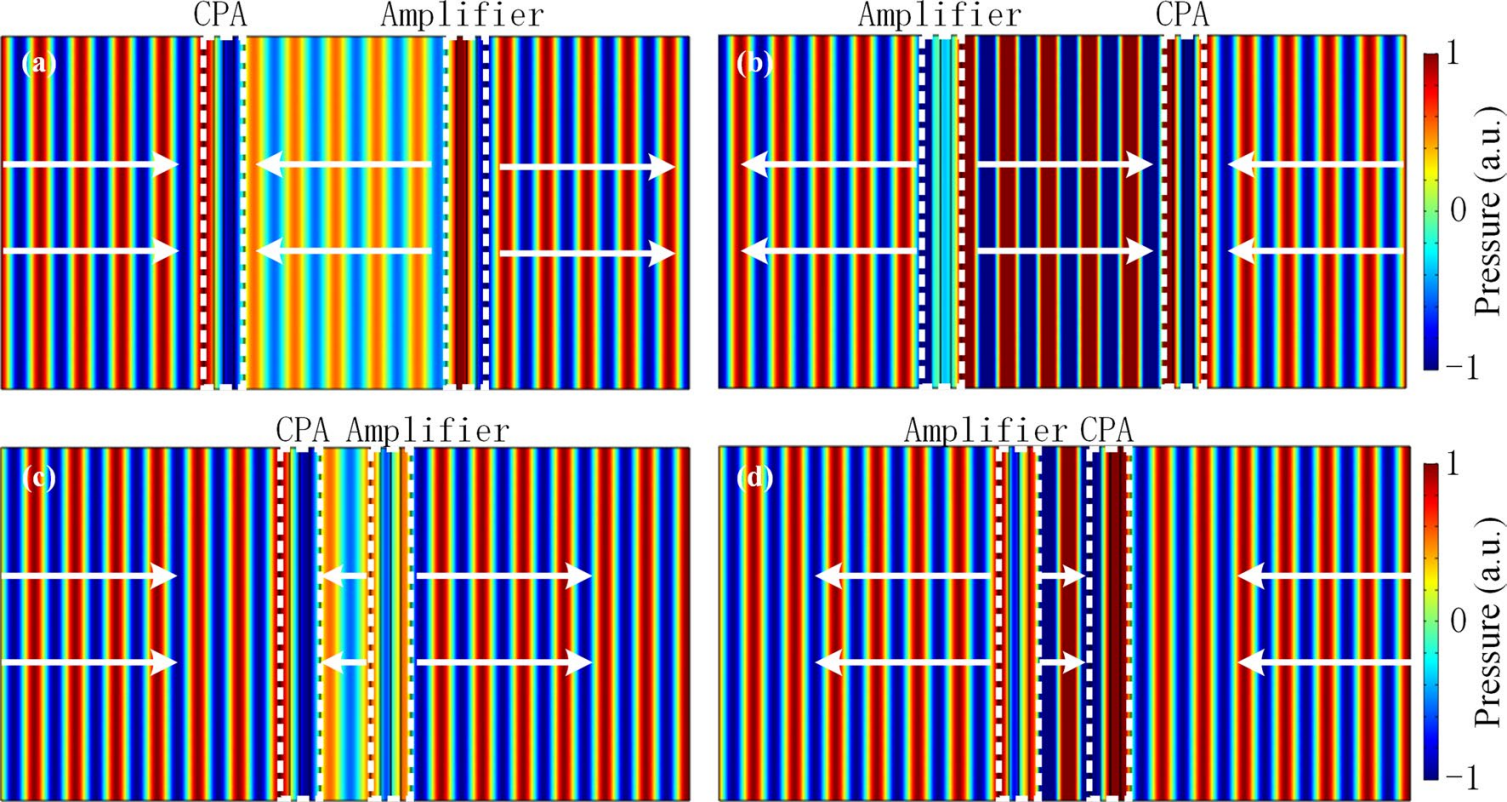
Pressure field distributions of the bidirectional acoustic negative refraction system for the normally incident plane wave at the operating frequency of *f* = 3,400 Hz. The distances between two metasurfaces are set to (**a**), (**b**) *d* = 500 mm and (**c**), (**d**) *d* = 125 mm, respectively. In (**a**) and (**c**), the plane wave is incident from the left side. In (**b**) and (**d**), the plane wave is incident from the right side.

To gain physical insight into this intriguing phenomenon, we have performed numerical full-wave simulations by using a finite element solver (COMSOL Multiphysics software) to verify the bidirectional acoustic negative refraction effect of the designed *PT*-symmetric system. The plane acoustic wave is normally incident with frequency located at the CPA-saser point of the locally *PT*-symmetric metasurface (i.e., *f* = 3,400 Hz). The acoustic densities and velocities of the loss and gain layers are set as $\rho_{l} = 1.21\,{\text{kg}}/{\text{m}}^{3}$,$\rho_{g} = 1.2{1}\,{\text{kg}}/{\text{m}}^{{3}}$, $c_{l} = 305.25 + 107.35i$ and $c_{g} = 305.25 - 107.35i$, respectively. Figure 4a, b show the simulated pressure field distributions of the systems under plane waves incident normally from the left and right sides, respectively, where the distance between two metasurfaces is *d* = 500 mm for both cases. The white arrows in figures represent the direction of the power flow. In the numerical simulations, the front and back boundaries are perfect absorbing boundaries. As predicted by the theoretical analysis, the system is non-reflecting for the acoustic wave incident from the left and right sides, and the transmission coefficients are all around $\left| t \right| \approx 1$. This indicates that the pair of metasurfaces has unique ability to switch the states between CPA-amplifier pair and amplifier-CPA pair flexibility according to the direction of incident wave. Since energy flux flows from the amplifier to the CPA, the acoustic wave between two metasurfaces are propagating in the direction opposite to that of the incident wave, which provides a *phase advance* to the transmitted wave. Moreover, in Fig. 4c,d, we replace the distance *d* = 125 mm and still obtain left- (right-) reflection coefficient $r_{L(R)} \to 1$ and transmission coefficient $t \approx 1$, which indicates that the perfect transmission is independent of the distance *d* between two metasurfaces. Hence, the acoustic perfect transmission is clearly observed for the normally incident plane wave and the globally *PT*-symmetric system has potential ability in bidirectional acoustic negative refraction.

**Figure 5:**
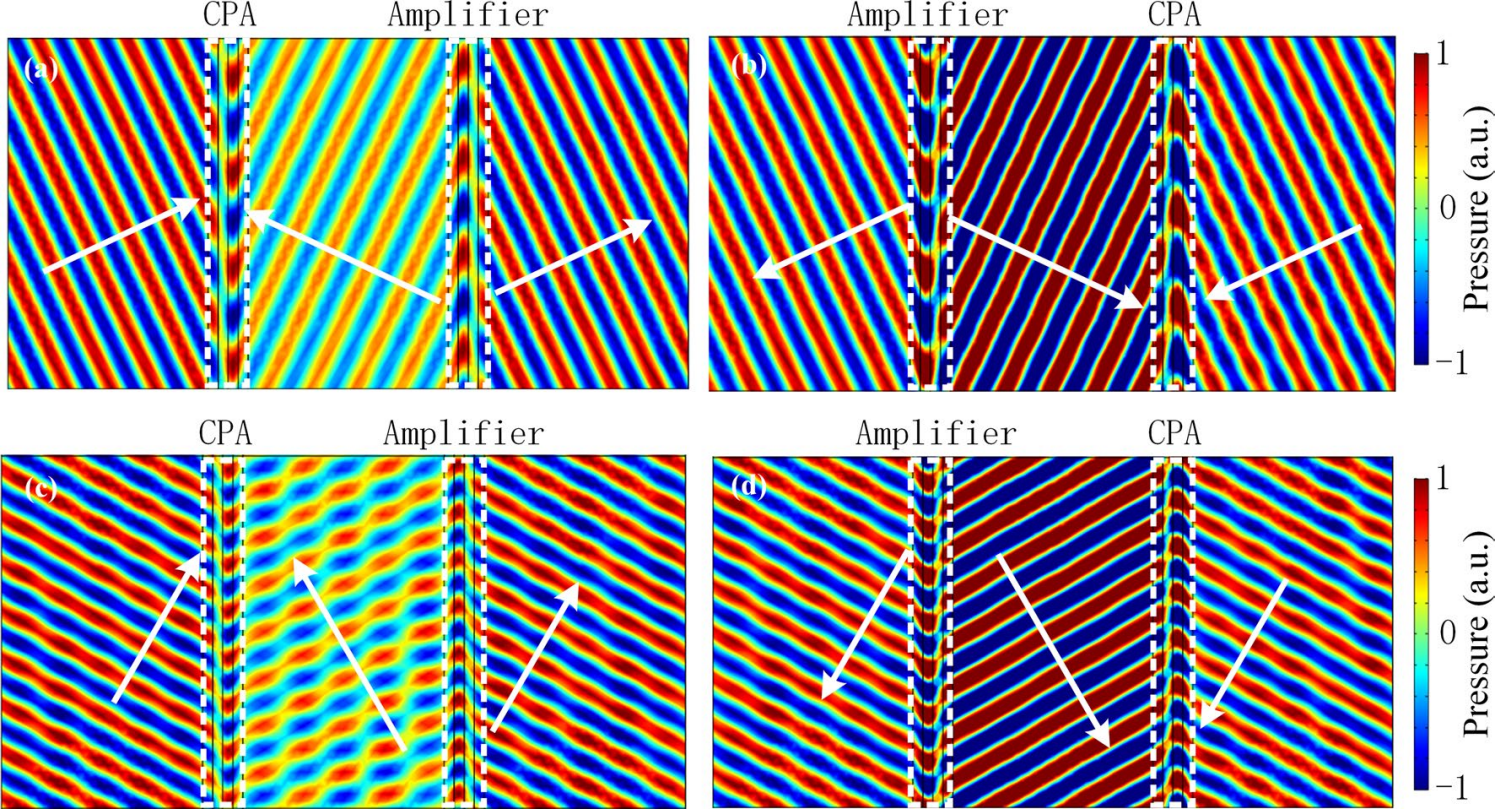
Pressure field distributions of the bidirectional acoustic negative refraction system for the obliquely incident plane wave at the operating frequency of *f* = 3,400 Hz. The angles of obliquely incident waves are set to (**a**), (**b**) $\theta = 25^{ \circ }$ and (**c**), (**d**) $\theta = 60^{ \circ }$, respectively. The white arrows represent the direction of the power flow. In (**a**) and (**c**), the plane wave is incident from the left side. In (**b**) and (**d**), the plane wave is incident from the right side.

It is important to note that this *PT*-symmetric system can be designed to negatively bend acoustic incident waves with any desired angle, as shown in Eq. (2). For the obliquely incident wave with angle $\theta$, to obtain the bidirectional negative refraction at the operating frequency 3,400 Hz, the acoustic densities and velocities of the loss and gain layers are set as $\rho_{l} (\theta ) = \rho_{l} /\cos \theta$, $\rho_{g} (\theta ) = \rho_{g} /\cos \theta$, $c_{l} (\theta ) = c_{l} \cos \theta_{l}$ and $c_{g} (\theta ) = c_{g} \cos \theta_{g}$, where $\theta_{l} = \arcsin (c_{l} (\theta )/c_{0} \sin \theta )$ and $\theta_{g} = \arcsin (c_{g} (\theta )/c_{0} \sin \theta )$ are the refracted angles in loss and gain layers, respectively. For $\theta = 25^{ \circ }$ and $\theta = 60^{ \circ }$, the pressure field distributions are presented in Fig. 5a–d, respectively. In Fig. 5a,c, the plane acoustic wave is incident from the left side. However, in Fig. 5b,d, the plane acoustic wave is incident from the right side. Here, the distance between two metasurfaces is set as *d* = 500 mm for both cases. Clearly, even for obliquely incident condition, the perfect transmission still exists in the system. The bidirectional acoustic negative refraction is clearly observed between two metasurfaces, where power transfers from the amplifier to the CPA. At the CPA-saser point, the pair of *PT*-symmetric metasurfaces still has unique ability to switch states between CPA-amplifier pair and amplifier-CPA pair simply by tuning the incident direction. Hence, this *PT*-symmetric system supports bidirectional acoustic negative refraction for obliquely incident wave.

**Figure 6:**
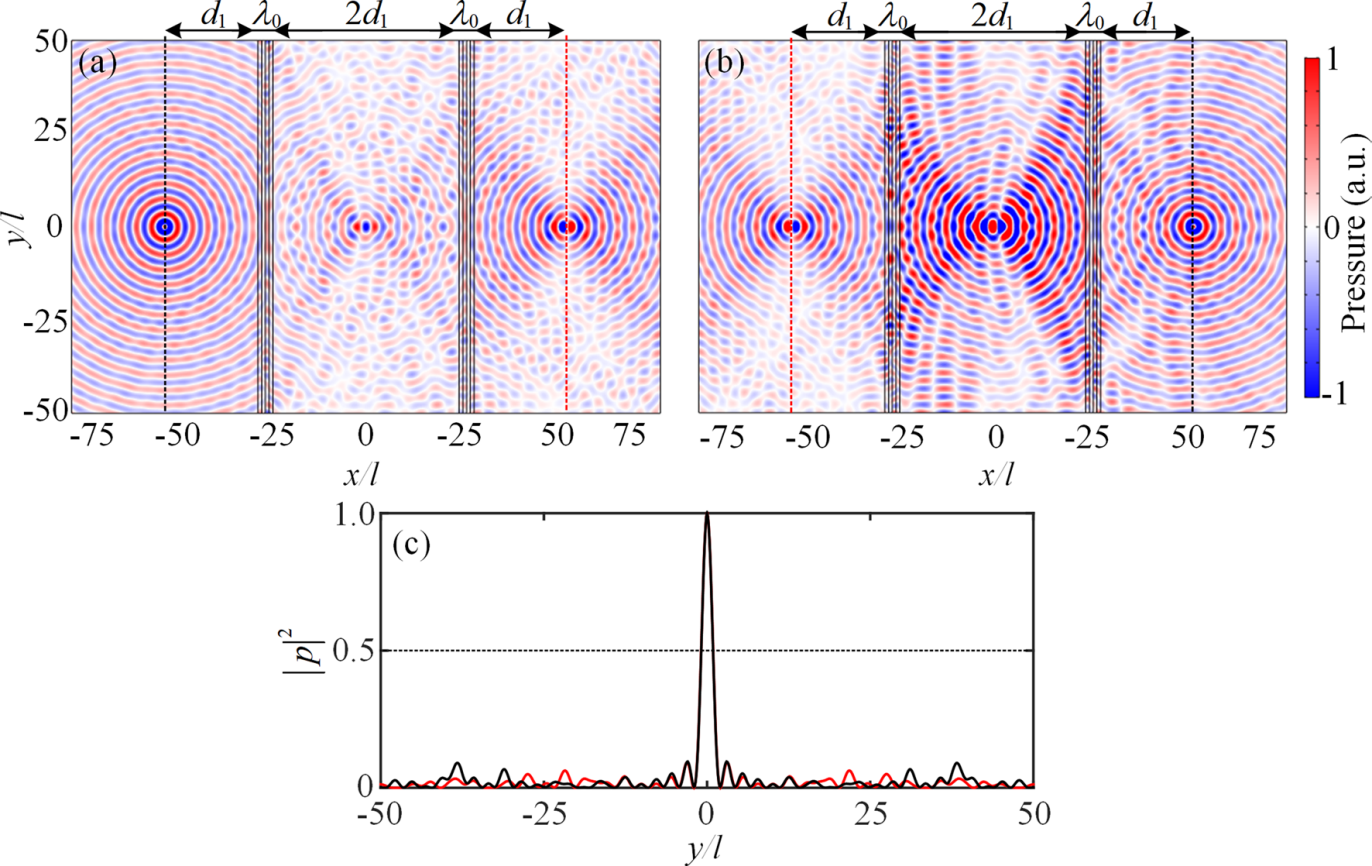
Pressure field distributions of the bidirectional acoustic planar focusing. The incident point sources are located on the (**a**) left and (**b**) right sides of the *PT*-symmetric system, respectively. (**c**) Red and black curves represent the intensity field on the focus planes shown in (**a**) and (**b**) with red dotted lines, respectively.

Since the *PT*-symmetric system has good resolution in both transverse and longitudinal directions, it has potential in ideally focusing the source with arbitrary angle^13^. If the incidence is a point source at an appropriate distance from the *PT*-symmetric system, it will again focus at two different distance points, which generates two images of the point source. Here, we assume the point source is placed at a distance *d*_1_ = 25*l* from the system on the left or right side, and the distance between two *PT*-symmetric metasurfaces is 2*d*_1_. The acoustic densities and velocities of the loss and gain layers along the longitudinal direction are variable, which are expressed as $\rho_{l} (\theta ) = \rho_{l} /\cos \theta$, $\rho_{g} (\theta ) = \rho_{g} /\cos \theta$, $c_{l} (\theta ) = c_{l} \cos \theta_{l}$ and $c_{g} (\theta ) = c_{g} \cos \theta_{g}$, where $\theta = \arctan (y/d_{1} )$ is dependent on the position in *y*-axis. Figure 6a,b show the simulated pressure field distributions of the proposed *PT*-symmetric system under point sources located on the left and right sides, respectively. As expected, two images of the point source are created at the center of two metasurfaces and the outside region of the system, respectively. Moreover, Fig. 6c shows the pressure intensity field on the focus plane at *d*_1_ distance from the *PT*-symmetry system. The half-power beam widths of the focus spots for both conditions of the point sources located on the left and right sides are all around $0.5\lambda_{0}$, which confirms that the proposed *PT*-symmetric system ideally focuses the propagating spectrum and offers good resolution. Therefore, the bidirectional *PT*-symmetric planar focusing is realized by the proposed *PT*-symmetric system.

The stability of the *PT*-symmetric system is a necessary issue, since the active parts is involved^12–14,24,29^. However, since the above discussions are limited to steady-state monochromatic operation, the stability does not necessary hold for other frequencies or for a realistic causal excitation. The detailed stability analysis of the proposed globally *PT*-symmetric system by applying a Lorentz dispersion model for the bulk modulus of the loss and gain layers is presented in Supplementary Information. The stability of *PT*-symmetric system is discussed by plotting the poles of the scattering matrix *S* on the complex frequency plane. It is found that it is always possible to ensure full stability for the proposed system by properly tailoring the frequency dispersion of the loss and gain layers.

Currently, the experimental study of acoustic *PT*-symmetric system is at an early stage. The challenge mostly lies in the design and realization of acoustic gain medium. In acoustics, for the one-dimensional two-port *PT*-symmetric system, the delicate feedback systems using the active-controlling apparatus have been widely applied to provide wave amplification in experiment^42–44^, although complex circuit and external energy are involved. Also, recently, a *PT*-symmetric metasurface cloaking system has also been experimentally realized using similar systems, which successfully achieved the functionality of unidirectional cloaking^45^. So far, other acoustic *PT*-symmetric systems have been mostly discussed in theory or constructed by the passive acoustic system without gain parts^40,46–48^. Therefore, the active parts in this work may require much more sophisticated design and engineering.

## Discussion

In conclusion, we have proposed a fundamental physical mechanism to change the general unidirectional behaviors in *PT*-symmetric systems into bidirectional behaviors. Such a mechanism involves the utilization of both local and global *PT*-symmetries. A pair of locally *PT*-symmetric multi-layer metasurfaces that sandwich a region of free space is used to design a globally *PT*-symmetric system. At the local CPA-saser point, this pair of metasurfaces can flexibly switch the states between CPA-amplifier pair and amplifier-CPA pair without resorting to altering the frequency or structure. This feature provides an approach to realizing bidirectional negative refraction with perfect transmission and planar focusing with good resolution. Our results may stimulate the potential of bidirectional characteristics in *PT*-symmetric systems and further prove the validity of using *PT* symmetry as a new method to overcome the limitations of passive system.

## Methods

Throughout the paper, the finite element method based on COMSOL Multiphysics software is employed for the numerical simulations. For Figs. 4, 5 and 6, the plane wave radiation boundaries are imposed on the incident and transmitted boundaries, and the periodic boundary conditions are employed in the *y* direction.

## Supplementary information

Supplementary file1 (PDF 1303 kb)
