# Supplementary material for: Bidirectional acoustic negative refraction based on a pair of metasurfaces with both local and global PT-symmetries
Source: Sci Rep. 2020 Jul 1;10:10794. doi: 10.1038/s41598-020-67793-x (PMC7330048; doi:10.1038/s41598-020-67793-x)
Supplement: Supplementary file 1 — Supplementary file1 (PDF 1303 kb) [file 41598_2020_67793_MOESM1_ESM.pdf]

<u>Supplementary Information for</u>

Bidirectional acoustic negative refraction based on a pair of metasurfaces with both local and global *PT*-symmetries

Jun Lan,[1] Xiaowei Zhang,[1] Liwei Wang,[1] Yun Lai,[2,*] and Xiaozhou Liu[1,*]

[1]*Key Laboratory of Modern Acoustics, Institute of Acoustics and School of Physics, Collaborative Innovation Center of Advanced Microstructures, Nanjing University, Nanjing 210093, P. R. China*

[2]*Key Laboratory of Modern Acoustics, National Laboratory of Solid State Microstructures, School of Physics, and Collaborative Innovation Center of Advanced Microstructures, Nanjing University, Nanjing 210093, P. R. China*

***Correspondence:** Xiaozhou Liu (email: xzliu@nju.edu.cn); Yun Lai (email: laiyun@nju.edu.cn)

1. Transfer matrix method

The globally *PT*-symmetric system is analyzed by the transfer matrix method. As shown in Supplementary Fig. S1, the globally *PT*-symmetric system is composed of eleven sections: four loss layers (*A*), four gain layers (*B*), and three free space regions (I, II, and III regions). When a plane acoustic wave is incident on the system, it will generate multiple transmissions and reflections that bound forth and back within each section. In Supplementary Fig. S1, we show the stationary forward- and backward-traveling waves in each section, which are the sum of an infinite number of transient forward- and backward-traveling waves, respectively.

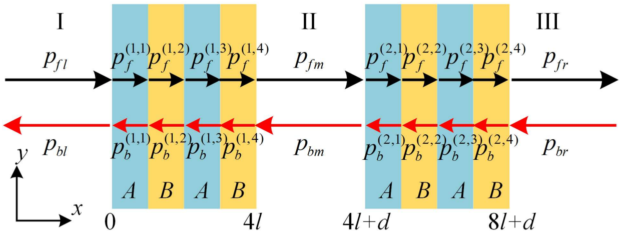

Supplementary Figure S1. Transfer matrix model of the globally *PT*-symmetric system.

When a plane acoustic wave is incident from the left side of the globally *PT*-symmetric system. We assume time-harmonic convention $e^{i\omega t}$, the expressions of the pressure field (p_{I}) and the particle velocity field (v_{I}) that contain forward- and backward-traveling waves in the I region ($x \le 0$) are given by

$$p_{\mathrm{I}} = p_{fl} e^{ik_0(x\cos\theta_f - y\sin\theta_f)} + p_{bl} e^{-ik_0(x\cos\theta_b + y\sin\theta_b)}, \tag{S1}$$

$$v_{\mathrm{I}} = \frac{\cos\theta_f}{\rho_0 c_0} p_{fl} e^{ik_0(x\cos\theta_f - y\sin\theta_f)} - \frac{\cos\theta_b}{\rho_0 c_0} p_{bl} e^{-ik_0(x\cos\theta_b + y\sin\theta_b)}, \tag{S2}$$

where ρ_0, c_0 and k_0 are the density, the speed and the wave number in free space, respectively. p_{fl} and p_{bl} are the components of the forward- and backward-traveling acoustic waves in the I region, respectively. θ_f is the angle between forward traveling wave and *x*-axis (incident angle), and θ_b is the angle between backward traveling wave and *x*-axis (reflected angle). The pressure field and the particle velocity

field that contain forward- and backward-traveling acoustic waves in the m-th layer of the left/right metasurface ((1, m)/(2, m), m = 1,2,3,4) are given by

$$p_{(1,m)} = p_f^{(1,m)} e^{-ik^{(1,m)}((x-(m-1)l)\cos\theta_f^{(1,m)}+y\sin\theta_f^{(1,m)})} + p_b^{(1,m)} e^{ik^{(1,m)}((x-(m-1)l)\cos\theta_b^{(1,m)}-y\sin\cos\theta_b^{(1,m)})}, \quad \text{(S3)}$$

$$v_{(1,m)} = \frac{\cos\theta_f^{(1,m)}}{\rho^{(1,m)}c^{(1,m)}} p_f^{(1,m)} e^{-ik^{(1,m)}((x-(m-1)l)\cos\theta_f^{(1,m)}+y\sin\theta_f^{(1,m)})} - \frac{\cos\theta_b^{(1,m)}}{\rho^{(1,m)}c^{(1,m)}} p_b^{(1,m)} e^{ik^{(1,m)}((x-(m-1)l)\cos\theta_b^{(1,m)}-y\sin\theta_b^{(1,m)})}, \quad \text{(S4)}$$

$$p_{(2,m)} = p_f^{(2,m)} e^{-ik^{(2,m)}((x-(3+m)l-d)\cos\theta_f^{(2,m)}+y\sin\theta_f^{(2,m)})} + p_b^{(2,m)} e^{ik^{(2,m)}((x-(3+m)l-d)\cos\theta_b^{(2,m)}-y\sin\theta_b^{(2,m)})}, \quad \text{(S5)}$$

$$v_{(2,m)} = \frac{\cos\theta_f^{(2,m)}}{\rho^{(2,m)}c^{(2,m)}} p_f^{(2,m)} e^{-ik^{(2,m)}((x-(3+m)l-d)\cos\theta_f^{(2,m)}+y\sin\theta_f^{(2,m)})} - \frac{\cos\theta_b^{(2,m)}}{\rho^{(2,m)}c^{(2,m)}} p_b^{(2,m)} e^{ik^{(2,m)}((x-(3+m)l-d)\cos\theta_b^{(2,m)}-y\sin\theta_b^{(2,m)})}, \quad \text{(S6)}$$

where $p_{(1,m)}$ and $v_{(1,m)}$ are the pressure and associated particle velocity fields in the m-th layer of the left metasurface, respectively. $p_{(2,m)}$ and $v_{(2,m)}$ are that in the m-th layer of the right metasurface, respectively. $\rho^{(1,m)}(\rho^{(2,m)})$, $c^{(1,m)}(c^{(2,m)})$ and $k^{(1,m)}(k^{(2,m)})$ are the density, the speed and the wave number in the m-th layer of the left (right) metasurface, respectively. $\theta_f^{(1,m)}$ or $\theta_f^{(2,m)}$ is the angle between forward-traveling wave and x-axis in the m-th layer of the left or right metasurface, respectively. $\theta_b^{(1,m)}$ or $\theta_b^{(2,m)}$ is the angle between backward-traveling wave and x-axis in the m-th layer of the left or right metasurface, respectively. The pressure field (p_{II}) and the particle velocity field (v_{II}) that contain forward- and backward-traveling waves in the II region ($4l<x<4l+d$) are given by

$$p_{\mathrm{II}} = p_{fm} e^{-ik_0((x-4l)\cos\theta_f+y\sin\theta_f)} + p_{bm} e^{ik_0((x-4l)\cos\theta_b-y\sin\theta_b)}, \quad \text{(S7)}$$

$$v_{\mathrm{II}} = \frac{\cos\theta_f}{\rho_0 c_0} p_{fm} e^{-ik_0((x-4l)\cos\theta_f+y\sin\theta_f)} - \frac{\cos\theta_b}{\rho_0 c_0} p_{bm} e^{ik_0((x-4l)\cos\theta_b-y\sin\theta_b)}, \quad \text{(S8)}$$

where p_{fm} and p_{bm} are the components of the forward- and backward-traveling acoustic waves in the II region, respectively. When a plane acoustic wave is incident from the left side of the globally PT-symmetric system, According to boundary

conditions, the pressures and particle velocities in the interface of two different media are continuous. The forward- and backward-traveling acoustic waves at both sides of the left metasurface are connected through the transfer matrix T_l, which can be expressed as

$$\begin{pmatrix} p_{fm} \\ p_{bm} \end{pmatrix} = T_l \begin{pmatrix} p_{fl} \\ p_{bl} \end{pmatrix}, \tag{S9}$$

where $T_l = T_{l4}T_{l3}T_{l2}T_{l1}T_{l0}$, in which

$$T_{l0} = \begin{bmatrix} \left(1+\frac{\rho^{(1,1)}c^{(1,1)}}{\rho_0 c_0}\frac{\cos\theta_f}{\cos\theta_f^{(1,1)}}\right)\Big/2 & \left(1-\frac{\rho^{(1,1)}c^{(1,1)}}{\rho_0 c_0}\frac{\cos\theta_f}{\cos\theta_f^{(1,1)}}\right)\Big/2 \\ \left(1-\frac{\rho^{(1,1)}c^{(1,1)}}{\rho_0 c_0}\frac{\cos\theta_f}{\cos\theta_f^{(1,1)}}\right)\Big/2 & \left(1+\frac{\rho^{(1,1)}c^{(1,1)}}{\rho_0 c_0}\frac{\cos\theta_f}{\cos\theta_f^{(1,1)}}\right)\Big/2 \end{bmatrix},$$

$$T_{lm} = M_{lm}N_{lm} = \begin{bmatrix} \left(1+\frac{\rho^{(1,m+1)}c^{(1,m+1)}}{\rho^{(1,m)}c^{(1,m)}}\frac{\cos\theta_f^{(1,m)}}{\cos\theta_f^{(1,m+1)}}\right)\Big/2 & \left(1-\frac{\rho^{(1,m+1)}c^{(1,m+1)}}{\rho^{(1,m)}c^{(1,m)}}\frac{\cos\theta_f^{(1,m)}}{\cos\theta_f^{(1,m+1)}}\right)\Big/2 \\ \left(1-\frac{\rho^{(1,m+1)}c^{(1,m+1)}}{\rho^{(1,m)}c^{(1,m)}}\frac{\cos\theta_f^{(1,m)}}{\cos\theta_f^{(1,m+1)}}\right)\Big/2 & \left(1+\frac{\rho^{(1,m+1)}c^{(1,m+1)}}{\rho^{(1,m)}c^{(1,m)}}\frac{\cos\theta_f^{(1,m)}}{\cos\theta_f^{(1,m+1)}}\right)\Big/2 \end{bmatrix}$$
$$\times \begin{bmatrix} e^{-ik^{(1,m)}l\cos\theta_f^{(1,m)}} & 0 \\ 0 & e^{ik^{(1,m)}l\cos\theta_f^{(1,m)}} \end{bmatrix} \quad m=1,2,3$$

$$T_{l4} = M_{l4}N_{l4} = \begin{bmatrix} \left(1+\frac{\rho_0 c_0}{\rho^{(1,4)}c^{(1,4)}}\frac{\cos\theta_f^{(1,4)}}{\cos\theta_f}\right)\Big/2 & \left(1-\frac{\rho_0 c_0}{\rho^{(1,4)}c^{(1,4)}}\frac{\cos\theta_f^{(1,4)}}{\cos\theta_f}\right)\Big/2 \\ \left(1-\frac{\rho_0 c_0}{\rho^{(1,4)}c^{(1,4)}}\frac{\cos\theta^{(1,4)}}{\cos\theta_f}\right)\Big/2 & \left(1+\frac{\rho_0 c_0}{\rho^{(1,4)}c^{(1,4)}}\frac{\cos\theta_f^{(1,4)}}{\cos\theta_f}\right)\Big/2 \end{bmatrix}$$
$$\times \begin{bmatrix} e^{-ik^{(1,4)}l\cos\theta_f^{(1,4)}} & 0 \\ 0 & e^{ik^{(1,4)}l\cos\theta_f^{(1,4)}} \end{bmatrix}.$$

It should be noted that, according to Snell's law, the angle of the forward-traveling wave is equal to the one of the backward-traveling wave, i.e., $\theta_f^{(1,m)} = \theta_b^{(1,m)}$, $\theta_f^{(2,m)} = \theta_b^{(2,m)}$ and $\theta_f = \theta_b$. The forward- and backward-traveling acoustic waves at both sides of the right metasurface are connected through the transfer matrix T_r, which can be expressed as

$$\begin{pmatrix} p_{fr} \\ p_{br} \end{pmatrix} = T_r \begin{pmatrix} p_{fm} \\ p_{bm} \end{pmatrix}, \tag{S10}$$

where $T_r = T_{r4}T_{r3}T_{r2}T_{r1}T_{r0}$, in which

$$T_{r0} = M_{r0}N_{r0} = \begin{bmatrix} \left(1+\dfrac{\rho^{(2,1)}c^{(2,1)}}{\rho_0 c_0}\dfrac{\cos\theta_f}{\cos\theta_f^{(2,1)}}\right)\Big/2 & \left(1-\dfrac{\rho^{(2,1)}c^{(2,1)}}{\rho_0 c_0}\dfrac{\cos\theta_f}{\cos\theta_f^{(2,1)}}\right)\Big/2 \\ \left(1-\dfrac{\rho^{(2,1)}c^{(2,1)}}{\rho_0 c_0}\dfrac{\cos\theta_f}{\cos\theta_f^{(2,1)}}\right)\Big/2 & \left(1+\dfrac{\rho^{(2,1)}c^{(2,1)}}{\rho_0 c_0}\dfrac{\cos\theta_f}{\cos\theta_f^{(2,1)}}\right)\Big/2 \end{bmatrix} \times \begin{bmatrix} e^{-ik_0 d\cos\theta_f} & 0 \\ 0 & e^{ik_0 d\cos\theta_f} \end{bmatrix},$$

$$T_{rm} = M_{rm}N_{rm} = \begin{bmatrix} \left(1+\dfrac{\rho^{(2,m+1)}c^{(2,m+1)}}{\rho^{(2,m)}c^{(2,m)}}\dfrac{\cos\theta_f^{(2,m)}}{\cos\theta_f^{(2,m+1)}}\right)\Big/2 & \left(1-\dfrac{\rho^{(2,m+1)}c^{(2,m+1)}}{\rho^{(2,m)}c^{(2,m)}}\dfrac{\cos\theta_f^{(2,m)}}{\cos\theta_f^{(2,m+1)}}\right)\Big/2 \\ \left(1-\dfrac{\rho^{(2,m+1)}c^{(2,m+1)}}{\rho^{(2,m)}c^{(2,m)}}\dfrac{\cos\theta_f^{(2,m)}}{\cos\theta_f^{(2,m+1)}}\right)\Big/2 & \left(1+\dfrac{\rho^{(2,m+1)}c^{(2,m+1)}}{\rho^{(2,m)}c^{(2,m)}}\dfrac{\cos\theta_f^{(2,m)}}{\cos\theta_f^{(2,m+1)}}\right)\Big/2 \end{bmatrix} \times \begin{bmatrix} e^{-ik^{(2,m)}l\cos\theta_f^{(2,m)}} & 0 \\ 0 & e^{ik^{(2,m)}l\cos\theta_f^{(2,m)}} \end{bmatrix}, \quad m=1,2,3$$

$$T_{r4} = M_{r4}N_{r4} = \begin{bmatrix} \left(1+\dfrac{\rho_0 c_0}{\rho^{(2,4)}c^{(2,4)}}\dfrac{\cos\theta_f^{(2,4)}}{\cos\theta_f}\right)\Big/2 & \left(1-\dfrac{\rho_0 c_0}{\rho^{(2,4)}c^{(2,4)}}\dfrac{\cos\theta_f^{(2,4)}}{\cos\theta_f}\right)\Big/2 \\ \left(1-\dfrac{\rho_0 c_0}{\rho^{(2,4)}c^{(2,4)}}\dfrac{\cos\theta_f^{(2,4)}}{\cos\theta_f}\right)\Big/2 & \left(1+\dfrac{\rho_0 c_0}{\rho^{(2,4)}c^{(2,4)}}\dfrac{\cos\theta_f^{(2,4)}}{\cos\theta_f}\right)\Big/2 \end{bmatrix} \begin{bmatrix} e^{-ik^{(2,4)}l\cos\theta_f^{(2,4)}} & 0 \\ 0 & e^{ik^{(2,4)}l\cos\theta_f^{(2,4)}} \end{bmatrix}.$$

Therefore, when a plane acoustic wave is incident from the left side of the globally *PT*-symmetric system, the forward- and backward-traveling acoustic waves at both sides of the *PT*-symmetric system are connected through the transfer matrix

$$\begin{pmatrix} p_{fr} \\ p_{br} \end{pmatrix} = T_r T_l \begin{pmatrix} p_{fl} \\ p_{bl} \end{pmatrix} = T_{r4}T_{r3}T_{r2}T_{r1}T_{r0}T_{l4}T_{l3}T_{l2}T_{l1}T_{l0} \begin{pmatrix} p_{fl} \\ p_{bl} \end{pmatrix}. \tag{S11}$$

In addition, when an acoustic wave is incident from the right side of the globally *PT*-symmetric system, the forward- and backward-traveling acoustic waves at both sides of the *PT*-symmetric system are connected through the transfer matrix

$$\begin{pmatrix} p_{fl} \\ p_{bl} \end{pmatrix} = T_l' T_r' \begin{pmatrix} p_{fr} \\ p_{br} \end{pmatrix} = T_{l1}' T_{l2}' T_{l3}' T_{l4}' T_{l0}' T_{r1}' T_{r2}' T_{r3}' T_{r4}' T_{r0}' \begin{pmatrix} p_{fr} \\ p_{br} \end{pmatrix}, \tag{S12}$$

where $\begin{pmatrix} p_{fm} \\ p_{bm} \end{pmatrix} = T_r' \begin{pmatrix} p_{fr} \\ p_{br} \end{pmatrix}$, $\begin{pmatrix} p_{fl} \\ p_{bl} \end{pmatrix} = T_l' \begin{pmatrix} p_{fm} \\ p_{bm} \end{pmatrix}$, $T_r' = T_{r1}' T_{r2}' T_{r3}' T_{r4}' T_{r0}'$, and $T_l' = T_{l1}' T_{l2}' T_{l3}' T_{l4}' T_{l0}'$, in which

$$T_{r0}' = \begin{bmatrix} \left(1 + \dfrac{\rho^{(2,4)} c^{(2,4)}}{\rho_0 c_0} \dfrac{\cos\theta_f}{\cos\theta_f^{(2,4)}}\right)\Big/2 & \left(1 - \dfrac{\rho^{(2,4)} c^{(2,4)}}{\rho_0 c_0} \dfrac{\cos\theta_f}{\cos\theta_f^{(2,4)}}\right)\Big/2 \\ \left(1 - \dfrac{\rho^{(2,4)} c^{(2,4)}}{\rho_0 c_0} \dfrac{\cos\theta_f}{\cos\theta_f^{(2,4)}}\right)\Big/2 & \left(1 + \dfrac{\rho^{(2,4)} c^{(2,4)}}{\rho_0 c_0} \dfrac{\cos\theta_f}{\cos\theta_f^{(2,4)}}\right)\Big/2 \end{bmatrix},$$

$$\begin{aligned} T_{r(m+1)}' &= M_{r(m+1)}' N_{r(m+1)}' \\ &= \begin{bmatrix} \left(1 + \dfrac{\rho^{(2,m)} c^{(2,m)}}{\rho^{(2,m+1)} c^{(2,m+1)}} \dfrac{\cos\theta_f^{(2,m+1)}}{\cos\theta_f^{(2,m)}}\right)\Big/2 & \left(1 - \dfrac{\rho^{(2,m)} c^{(2,m)}}{\rho^{(2,m+1)} c^{(2,m+1)}} \dfrac{\cos\theta_f^{(2,m+1)}}{\cos\theta_f^{(2,m)}}\right)\Big/2 \\ \left(1 - \dfrac{\rho^{(2,m)} c^{(2,m)}}{\rho^{(2,m+1)} c^{(2,m+1)}} \dfrac{\cos\theta_f^{(2,m+1)}}{\cos\theta_f^{(2,m)}}\right)\Big/2 & \left(1 + \dfrac{\rho^{(2,m)} c^{(2,m)}}{\rho^{(2,m+1)} c^{(2,m+1)}} \dfrac{\cos\theta_f^{(2,m+1)}}{\cos\theta_f^{(2,m)}}\right)\Big/2 \end{bmatrix} \\ &\times \begin{bmatrix} e^{-ik^{(2,m+1)} l \cos\theta_f^{(2,m+1)}} & 0 \\ 0 & e^{ik^{(2,m+1)} l \cos\theta_f^{(2,m+1)}} \end{bmatrix}, \quad m = 3,2,1 \end{aligned}$$

$$\begin{aligned} T_{r1}' &= M_{r1}' N_{r1}' \\ &= \begin{bmatrix} \left(1 + \dfrac{\rho_0 c_0}{\rho^{(2,1)} c^{(2,1)}} \dfrac{\cos\theta_f^{(2,1)}}{\cos\theta_f}\right)\Big/2 & \left(1 - \dfrac{\rho_0 c_0}{\rho^{(2,1)} c^{(2,1)}} \dfrac{\cos\theta_f^{(2,1)}}{\cos\theta_f}\right)\Big/2 \\ \left(1 - \dfrac{\rho_0 c_0}{\rho^{(2,1)} c^{(2,1)}} \dfrac{\cos\theta_f^{(2,1)}}{\cos\theta_f}\right)\Big/2 & \left(1 + \dfrac{\rho_0 c_0}{\rho^{(2,1)} c^{(2,1)}} \dfrac{\cos\theta_f^{(2,1)}}{\cos\theta_f}\right)\Big/2 \end{bmatrix} \begin{bmatrix} e^{-ik^{(2,1)} l \cos\theta_f^{(2,1)}} & 0 \\ 0 & e^{ik^{(2,1)} l \cos\theta_f^{(2,1)}} \end{bmatrix}, \end{aligned}$$

$$\begin{aligned} T_{l0}' = M_{l0}' N_{l0}' &= \begin{bmatrix} \left(1 + \dfrac{\rho^{(1,4)} c^{(1,4)}}{\rho_0 c_0} \dfrac{\cos\theta_f}{\cos\theta_f^{(1,4)}}\right)\Big/2 & \left(1 - \dfrac{\rho^{(1,4)} c^{(1,4)}}{\rho_0 c_0} \dfrac{\cos\theta_f}{\cos\theta_f^{(1,4)}}\right)\Big/2 \\ \left(1 - \dfrac{\rho^{(1,4)} c^{(1,4)}}{\rho_0 c_0} \dfrac{\cos\theta_f}{\cos\theta_f^{(1,4)}}\right)\Big/2 & \left(1 + \dfrac{\rho^{(1,4)} c^{(1,4)}}{\rho_0 c_0} \dfrac{\cos\theta_f}{\cos\theta_f^{(1,4)}}\right)\Big/2 \end{bmatrix} \\ &\times \begin{bmatrix} e^{-ik_0 d \cos\theta_f} & 0 \\ 0 & e^{ik_0 d \cos\theta_f} \end{bmatrix}, \end{aligned}$$

$$T'_{l(m+1)} = M'_{l(m+1)} N'_{l(m+1)}$$

$$= \begin{bmatrix} \left(1+\frac{\rho^{(1,m)}c^{(1,m)}}{\rho^{(1,m+1)}c^{(1,m+1)}}\frac{\cos\theta_f^{(1,m+1)}}{\cos\theta_f^{(1,m)}}\right)\Big/2 & \left(1-\frac{\rho^{(1,m)}c^{(1,m)}}{\rho^{(1,m+1)}c^{(1,m+1)}}\frac{\cos\theta_f^{(1,m+1)}}{\cos\theta_f^{(1,m)}}\right)\Big/2 \\ \left(1-\frac{\rho^{(1,m)}c^{(1,m)}}{\rho^{(1,m+1)}c^{(1,m+1)}}\frac{\cos\theta_f^{(1,m+1)}}{\cos\theta_f^{(1,m)}}\right)\Big/2 & \left(1+\frac{\rho^{(1,m)}c^{(1,m)}}{\rho^{(1,m+1)}c^{(1,m+1)}}\frac{\cos\theta_f^{(1,m+1)}}{\cos\theta_f^{(1,m)}}\right)\Big/2 \end{bmatrix}$$

$$\times \begin{bmatrix} e^{-ik^{(1,m+1)}l\cos\theta_f^{(1,m+1)}} & 0 \\ 0 & e^{ik^{(1,m+1)}l\cos\theta_f^{(1,m+1)}} \end{bmatrix}, \quad m = 3,2,1$$

$$T'_{l1} = M'_{l1} N'_{l1}$$

$$= \begin{bmatrix} \left(1+\frac{\rho_0 c_0}{\rho^{(1,1)}c^{(1,1)}}\frac{\cos\theta_f^{(1,1)}}{\cos\theta_f}\right)\Big/2 & \left(1-\frac{\rho_0 c_0}{\rho^{(1,1)}c^{(1,1)}}\frac{\cos\theta_f^{(1,1)}}{\cos\theta_f}\right)\Big/2 \\ \left(1-\frac{\rho_0 c_0}{\rho^{(1,1)}c^{(1,1)}}\frac{\cos\theta_f^{(1,1)}}{\cos\theta_f}\right)\Big/2 & \left(1+\frac{\rho_0 c_0}{\rho^{(1,1)}c^{(1,1)}}\frac{\cos\theta_f^{(1,1)}}{\cos\theta_f}\right)\Big/2 \end{bmatrix} \begin{bmatrix} e^{-ik^{(1,1)}l\cos\theta_f^{(1,1)}} & 0 \\ 0 & e^{ik^{(1,1)}l\cos\theta_f^{(1,1)}} \end{bmatrix}.$$

The reflection coefficients for the acoustic waves incident from the left side (r_L) and the right side (r_R) of the globally *PT*-symmetric system can be obtained from Eqs. (S11) and (S12), respectively. The transmission coefficients (*t*) under the left and right incidences calculated from Eqs. (S11) and (S12) are the same due to reciprocity.

2. Stability of the globally *PT*-symmetric system

In the main text, we have considered the globally *PT*-symmetric system excited by purely monochromatic source at steady states and stability does not necessary hold for other frequencies. Here, a full investigation of the stability of the globally *PT*-symmetric system is discussed. In practice, the stability of the system is determined by the frequency dispersion of the material properties of the loss and gain parts [1-5]. A conventional Lorentz dispersion model is assumed to describe the relative bulk modulus ($\kappa_{ms,l}$) of the loss layer (*A*) as

$$\frac{1}{\kappa_{ms,l}} = \frac{1}{\kappa_{\infty,l}} + \frac{\omega_{p,l}^2}{\omega_{0,l}^2 + i\omega\gamma_l - \omega^2}, \tag{S13}$$

and an anti-Lorentz dispersion model of the relative bulk modulus ($\kappa_{ms,g}$) for the gain layer (*B*) is assumed as

$$\frac{1}{\kappa_{ms,g}}=\frac{1}{\kappa_{\infty,g}}-\frac{\omega_{p,g}^2}{\omega_{0,g}^2+i\omega\gamma_g-\omega^2}, \tag{S14}$$

where ω_p, ω_0 and γ indicate the plasma frequency, resonance frequency and collision frequency of the medium, respectively, and the subscript *l* and *g* represent the loss layer and gain layer, respectively.

In the main text, we have $c_l \approx 305.25+107.35i$ and $c_g \approx 305.25-107.35i$, obtained for the normally incident wave with operating frequency 3400 Hz. Thus the refractive indices of the loss and gain layers can be calculated as $n_l = c_0/c_l = c_0\Big/\sqrt{\kappa_{ms,l}/\rho_l}$ and $n_g = c_0/c_g = c_0\Big/\sqrt{\kappa_{ms,g}/\rho_g}$. To fulfill the relative bulk modulus of the loss and gain layers at the central frequency 3400 Hz, the related parameters of the loss and gain layers in Eqs. (S13) and (S14) are set to be $\kappa_{\infty,l}=\kappa_{\infty,g}=1.4133\times10^5$, $\omega_{0,l}=0.999\omega_c$, $\omega_{0,g}=1.001\omega_c$, $\omega_{p,l}=\omega_{p,g}=5.0053$ Hz and $\gamma_l=\gamma_g=229.4057$ Hz, where $\omega_c=2\pi3400$ Hz is the center frequency of the operation of the globally *PT*-symmetric system. Supplementary Fig. S2(a) shows the real and imaginary parts of the bulk moduli of the loss and gain layers as functions of frequency. Supplementary Fig. S2(b) shows the poles of the scattering matrix *S* of the globally *PT*-symmetric system on the complex frequency plane, and the distance between two locally *PT*-symmetric multi-layer metasurfaces is *d* = 125 mm. It is seen that, all the poles lie in the upper half-plane, which indicates that the system is unconditionally stable for an arbitrary temporal excitation (since a $e^{i\omega t}$ time-harmonic convention is assumed here, poles in the lower half-plane corresponds to natural oscillations of the system that grow in time, hence instability). It is important to note that for different angles and different parameters choices, the system will become unstable and some of these poles lie in the lower half-plane, stability should be assessed in each case. However, through choosing the appropriate values of κ_∞, ω_p, ω_0 and γ in Eqs. (S13) and (S14), it is always possible to ensure full stability of the system. These above results indicate that such a *PT*-symmetric system can be made

unconditionally stable by properly tailoring the frequency dispersion of the loss and gain layers.

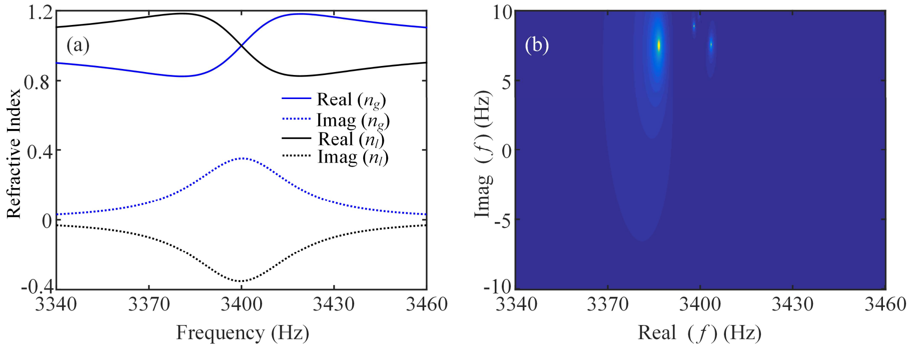

Supplementary Figure S2. (a) Frequency dispersion of the real (solid curves) and imaginary (dashed curves) parts of the relative refractive index of the loss and gain layers. (b) Poles of the scattering matrix *S* of the globally *PT*-symmetric system on the complex frequency plane.

References

1. Fleury, R., Sounas, D. L. & Alù, A. Negative refraction and planar focusing based on parity-time symmetric metasurfaces. *Phys. Rev. Lett.* **113**, 23903 (2014).

2. Sounas, D. L., Fleury, R. & Alù, A. Unidirectional cloaking based on metasurfaces with balanced loss and gain. *Phys. Rev. Appl.* **4**, 14005 (2015).

3. Valagainnopoulos, C. A., Monticone, F. & Alu, A. PT-symmetric planar devices for field transformation and imaging. *J. Optics-Uk* **18**, 44028 (2016).

4. Monticone, F., Valagiannopoulos, C. A. & Alù, A. Parity-time symmetric nonlocal metasurfaces: all-angle negative refraction and volumetric imaging. *Phys. Rev. X* **6**, 41018 (2016).

5. Luo, J., Li, J. & Lai, Y. Electromagnetic impurity-immunity induced by parity-time symmetry. *Phys. Rev. X* **8**, 031035 (2018).
